# Hook plate with or without coracoclavicular ligament augmentation in the treatment of acute acromioclavicular separation

**DOI:** 10.1186/s12891-020-03726-z

**Published:** 2020-10-23

**Authors:** Chung-Ting Liu, Ten-Fang Yang

**Affiliations:** 1 Biomedical Science and Engineering, National Chiao Tung University, No. 75, Bo’ai St., East Dist., Hsinchu City, 300 Taiwan, Republic of China; 2 Department of Orthopaedics, Mackay Memorial Hospital, Taipei, Taiwan; 3 Department of Biological Science and Technology, National Chiao-Tung University, Hsinchu, Taiwan; 4 Graduate Institute of Medical Informatics, Taipei Medical University and Hospital, Taipei, Taiwan

**Keywords:** Acromioclavicular separation, Hook plate, Coracoclavicular ligament, Augmentation

## Abstract

**Background:**

Acromioclavicular (AC) separation can be treated with the use of a hook plate. Some studies have reported that coracoclavicular (CC) ligament augmentation is necessary to reduce the complications of hook plate fixation, whereas others recommend hook plate fixation alone without augmentation. The aim of this study was to compare the results and complications between these two groups.

**Methods:**

This was an observational case-control study. Patients with acute (less than 2 weeks) Rockwood type V AC separation were treated with a hook plate at our hospital. A total of 105 cases received hook plate fixation with CC ligament augmentation (group I), and 112 cases received hook plate fixation without augmentation (group II). Constant-Murley scores were used to evaluate the function before and after implant removal, and radiographs were taken to evaluate the complications. The results and complications were compared between groups.

**Results:**

Before removal, the Constant-Murley score was significantly higher in group I (mean, 50.1) than in group II (mean, 42.6) (*p* = 0.004); however, there was no significant difference between groups at 3 and 6 months after removal. The incidence of significant acromion osteolysis was higher in group II (65/112) than in group I (25/105). Before removal, the patients with significant acromion osteolysis had worse Constant-Murley scores than those of the patients without osteolysis in both groups. The incidence of peri-implant fracture of the hook plate was higher in group II (8/112) than in group I (1/105).

**Conclusion:**

The patients without CC ligament augmentation had worse functional results before hook plate removal, a higher incidence of radiographic acromion osteolysis, and a higher incidence of peri-implant fractures than those patients with CC ligament augmentation. Therefore, CC ligament augmentation is highly recommended to improve short-term outcomes and decrease complications for Rockwood type V AC separation treated by hook plate.

## Background

Acromioclavicular (AC) separation occurs as a result of a downward force being applied to the superior part of the acromion. Trauma to the shoulder affects the ligaments between the clavicle and scapula, and the acromion of the scapula is connected to the clavicle by the AC ligament [1]. The coracoclavicular (CC) ligaments connect the clavicle to the coracoid process, and the trapezoid and conoid ligaments form the CC ligaments [1]. AC joint injuries are most commonly classified using the six grade system described by Rockwood, which takes into account not only the AC joint itself, but also the CC ligament and the direction of clavicle dislocation with respect to the acromion. Type V is a more severe form, which is characterized by a 2- to 3-fold increase in the CC distance; furthermore, the shoulder has a severe droop secondary to downward displacement of the scapula due to loss of the clavicular strut, and such injuries generally require surgery [2].

There are many surgical options for the treatment of type V AC separation, and each has pros and cons. Hook plate fixation generally requires a second surgery for plate removal, but it allows for firm fixation and early shoulder mobility. Hook plate fixation has been reported as a safe and effective option to treat AC separation [3–15]. However, whether additional CC ligament augmentation should be applied with hook plate fixation for type V AC separation remains controversial. Some studies have reported that CC ligament augmentation is necessary to reduce the complications of hook plate fixation [16–20], whereas others recommend hook plate fixation alone without CC ligament augmentation to minimize soft tissue damage [21–23]. Therefore, the aim of this study was to compare the clinical and radiological results between patients of type V AC separation treated by hook plate fixation, with or without CC ligament augmentation.

## Methods

This study was a retrospective observational case-control study, and approval was received from the Ethics Committee of our institute. We reviewed the patients with AC separation treated with a hook plate from 2008 to 2018. Inclusion criteria were acute AC separation (less than 2 weeks), Rockwood type V AC separation, and duration of follow up more than 12 months. Exclusion criteria were delayed removal of the hook plate (more than 6 months), existing rotator cuff lesions, and multiple trauma. Four radiographs were taken to diagnose AC separation and evaluate the concomitant injuries, including chest posteroanterior (PA) view with the image including bilateral shoulder joints for comparison; shoulder anteroposterior (AP) view; shoulder oblique view; and scapula Y view. All patients were treated with a hook plate, and part of the enrolled patients underwent CC ligament augmentation (group I, *n* = 105), while the remaining patients had no augmentation (group II, *n* = 112). Whether patients received CC augmentation or not depended on the surgeon’s preference. The average age of the included patients was 50 (range, 18 to 62) in group I and 46 (range, 19 to 75) in group II.

Constant-Murley scores were used to evaluate the functional performance of the shoulder before hook plate removal, 3 months after removal, and 6 months after removal. This scoring system includes individual parameters, and provides an overall clinical functional assessment. The parameters included in the score are pain, activity level, arm positioning, strength of abduction, and range of motion (ROM). A full ROM score is 40 points, with 10 points each in external rotation, internal rotation, forward flexion, and lateral elevation [24, 25]. The Visual Analogue Scale (VAS) score was also used for evaluation and comparison.

In both groups, radiographs were taken before hook plate removal, 3 months after removal, and 6 months after removal. The distance from the superior border of the coracoid process to the inferior border of the clavicle was measured on standard shoulder AP view, and compared before and after implant removal in order to determine further displacement after removal. Significant further displacement after implant removal was defined as an increase in CC distance more than 5 mm. Radiographic acromion osteolysis, acromion fracture, and clavicle fracture were also compared between groups. Significant osteolysis was defined as depression of bone erosion more than 2 mm.

The difference in Constant-Murley scores between groups were compared using an independent *t*-test, and the incidence of radiographic acromion osteolysis was compared between groups by Chi-square test. Statistical significance was defined as 0.05, and a *p*-value < 0.05 was defined as a statistically significant difference.

CC ligament augmentation was performed using 6-mm nylon tape. A wire loop was first passed beneath the coracoid process by a wire passer with minimal invasion, and the deltoid muscle was preserved as much as possible. The location of the torn stump of the CC ligament on the clavicle was identified, and a hole was drilled in it. The tape was passed through the hole of the clavicle, and then passed around the coracoid process by the wire loop guide. After the hook plate was applied to reduce the AC separation, nylon tape was tied anterior to the clavicle to resemble the function of the CC ligament. If the clavicle was forced to anterior, it could be directly observed.

A hook plate system (Depuy Synthes, Chester, Pennsylvania, United States) with an 18-mm hook depth and 85-mm plate length was used in all cases. Passive motion of the shoulder joint was encouraged immediately after the surgery to reduce shoulder adhesion. In addition, sling protection was suggested for 4 weeks, but for no more than 6 weeks. Active motion of the shoulder joint was started after 4–6 weeks, but heavy lifting and weight bearing was not allowed until 12 weeks. Then, the second surgery for implant removal was discussed, and arranged as soon as possible. The average duration from hook plate fixation to removal was 101.3 days (range, 86 to 118) in group I and 102.8 days (range, 89 to 116) in group II. In group I, the hook plate was removed, but the CC ligament augmentation was left in place. Immediately following implant removal, all patients started to perform passive and active stretching exercises without restriction; however, heavy lifting and weight bearing was not allowed until after 12 weeks.

## Results

There were 105 cases in group I and 112 cases in group II. The average age of the patients was 50 (range, 18 to 62) years in group I and 46 (range, 19 to 75) years in group II. There was no significant difference in age between groups (*p* = 0.47).

The average duration from hook plate fixation to removal was 101.3 days (range, 86 to 118) days in group I and 102.8 (range, 89 to 116) days in group II. There was no significant difference in the timing of removal between groups (*p* = 0.85).

**Fig. 1:**
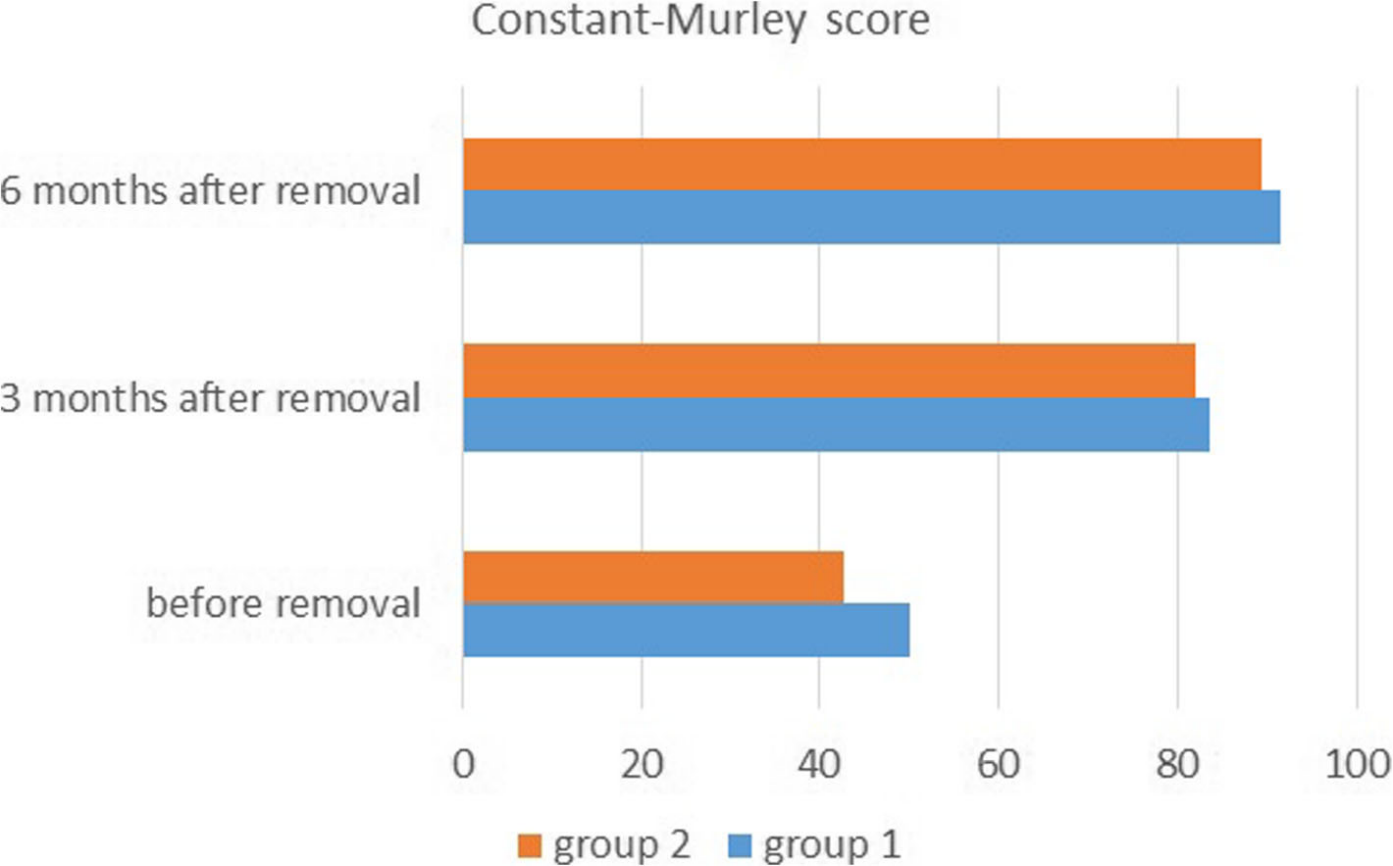
The Constant-Murley scores were significantly higher in group I than in group II before hook plate removal. However, there was no significant difference between groups at 3 and 6 months after removal. The scores of patients 3 months after removal were significantly higher than the scores before removal in both groups. Moreover, the scores of patients 6 months after removal was even higher than the scores 3 months after removal

Before removal, the average Constant-Murley score was 50.1 (range, 38 to 65; standard deviation, 3.32) in group I and 42.6 (range, 32 to 56; standard deviation, 3.93) in group II, with a significant difference between groups (*p* = 0.004) (Fig. 1). The average ROM score (full score 40) was 24.1 (range, 18 to 32) in group I and 22.7 (range, 16 to 32) in group II, with no significant difference between groups (*p* = 0.27). The average VAS score was 2.4 (range, 1 to 5) in group I and 3.1 (range, 1 to 5) in group II, with a significant difference between groups (*p* = 0.032).

Three months after removal, the average Constant-Murley score was 83.6 (range, 72 to 92; standard deviation, 7.62) in group I and 82.1 (range, 70 to 90; standard deviation, 7.89) in group II, with no significant difference between groups (*p* = 0.69) (Fig. 1). The average ROM score was 32.3 (range, 26 to 38) in group I and 33.2 (range, 24 to 38) in group II, with no significant difference between groups (*p* = 0.83). The average VAS score was 2.1 (range, 1 to 4) in group I and 2.0 (range, 1 to 5) in group II, with no significant difference between groups (*p* = 0.81).

Six months after removal, the average Constant-Murley score was 91.5 (range, 82 to 100; standard deviation, 5.56) in group I and 89.5 (range, 76 to 100; standard deviation, 6.12) in group II, with no significant difference between groups (*p* = 0.57) (Fig. 1). The average ROM score was 36.3 (range, 28 to 40) in group I and 34.7 (range, 26 to 40) in group II, with no significant difference between groups (*p* = 0.17). The average VAS score was 1.8 (range, 1 to 3) in group I and 1.9 (range, 1 to 3) in group II, with no significant difference between groups (*p* = 0.72).

The Constant-Murley score 3 months after removal was significantly higher than the score before removal in both groups (*p =* 0.0005 in group I and 0.0003 in group II) (Fig. 1), and the score 6 months after removal was even higher than the score 3 months after removal in both groups (*p* = 0.024 in group I and 0.028 in group II).

The VAS score 3 months after removal was lower than the score before removal, with a significant difference in both groups (*p* = 0.004 in group I and 0.009 in group II); however, there was no significant difference from 3 months to 6 months after removal (*p* = 0.08 in group I and 0.11 in group II).

To find further displacement after hook plate removal, the distance from the superior border of the coracoid process to the inferior border of the clavicle was measured on a standard shoulder AP view before and 3 months after removal. A total of 5 cases in group I and 8 cases in group II had further displacement of more than 5 mm on radiography after implant removal; however, these patients did not have worse Constant-Murley scores or any discomfort at the final follow-up.

**Fig. 2:**
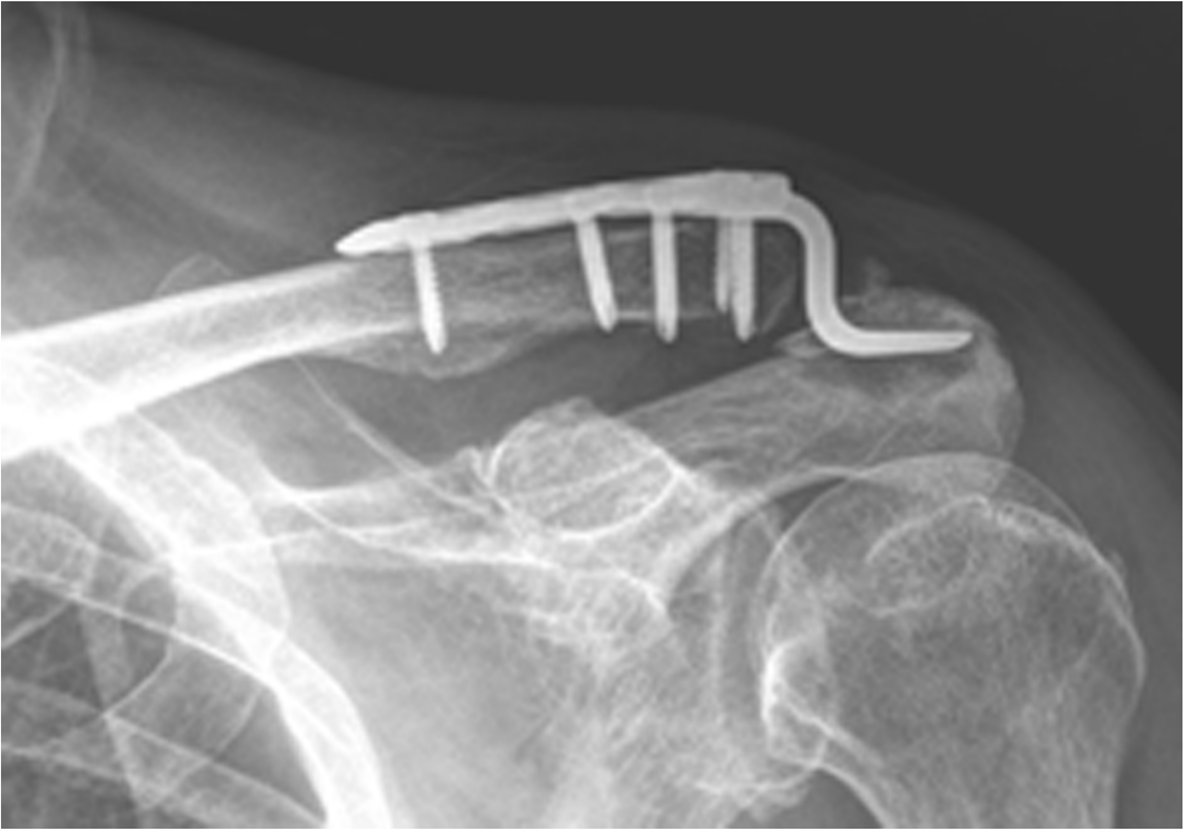
Acromion osteolysis was noted in both groups. The incidence of significant acromion osteolysis before removal was significantly higher in group II than in group I

Significant acromion osteolysis on radiography was noted before hook plate removal in 25 cases in group 1 (Fig. 2) and 65 cases in group 2. The incidence of significant acromion osteolysis was higher in group II (65/112) than in group I (25/105). Before removal, the patients with significant acromion osteolysis had worse Constant-Murley scores than those of the patients without osteolysis in both groups (*p* = 0.03 in group I and *p* = 0.04 in group II). Three months after removal and 6 months after removal, there was no significant difference in Constant-Murley scores between patients with and without osteolysis in both groups (*p* = 0.76 and 0.62 in group I; *p* = 0.85 and 0.73 in group II, respectively).

In group I, two patients had superficial wound infections and one patient had a distal clavicle fracture. In group II, seven patients had acromion linear fractures and one had a distal clavicle fracture. The incidence of peri-implant fracture of the hook plate was higher in group II (8/112) than in group I (1/105). There were no instances of broken implants, pneumothorax, great vessel injury, or other major complications in either group.

Patients without CC ligament augmentation had worse functional results before hook plate removal, a higher incidence of radiographic acromion osteolysis, and a higher incidence of peri-implant fracture.

## Discussion

The patients of Rockwood type V AC separation treated by hook plate without CC ligament augmentation had similar functional results after implant removal as those of the patients treated by hook plate with CC ligament augmentation. However, patients without CC ligament augmentation had worse functional results before hook plate removal, a higher incidence of radiographic acromion osteolysis, and a higher incidence of peri-implant fracture.

Many studies support the use of a hook plate to treat type V AC separations because it is considered to be safe, simple, effective, and reliable [3–15]. Loading tests were performed, and the results revealed that bending and torsional stiffness were significantly higher in clavicles fixed with a hook plate than those fitted with other methods [5, 26]. Thus, the use of a hook plate to treat type V AC separation has become increasingly popular in the past decade [27]. However, the hook plate requires a secondary operation to remove the plate, and delayed removal increases the incidence of associated problems such as osteolysis, frozen shoulder, or rotator cuff tear. Previous studies have reported that retaining a hook plate for more than 5 months results in a higher incidence of osteolysis and lower functional scores [28, 29]. Although there are no definite conclusions about the optimal timing of hook plate removal, it is generally preferred to remove the plate as soon as possible after ligamentous healing has been achieved [30]. Indeed, many surgeons prefer to perform CC ligament augmentation with hook plate fixation for type V AC separation to prevent delayed removal. However, AC separation treated by hook plate with and without CC ligament augmentation remains controversial.

Some surgeons perform CC ligament augmentation routinely with a hook plate because of the belief that additional CC ligament augmentation can improve the stability of the AC joint and prevent the recurrence of dislocation [16–20]. Furthermore, some studies have supported treating type V AC separation with a modified Weaver-Dunn procedure along with a hook plate to decrease the complication rate [31]. The Weaver-Dunn procedure is performed by replacing the CC ligament with a coracoacromial ligament [32].

Alternatively, other surgeons prefer to perform hook plate fixation alone without CC ligament augmentation. CC ligament augmentation requires a larger surgical wound, longer surgical time, more extensive soft tissue damage, and greater blood supply deprivation. In the current study, the post-removal Constant-Murley score revealed no significant difference in shoulder function between the two groups. A previous study reported similar results with a post-removal Constant Shoulder Score of 89 +/− 5 with hook plate fixation alone for type V AC separation; in this study, the authors recommended hook plate fixation without CC ligament augmentation [21]. Moreover, other studies also indicated that a hook plate alone can ensure a strong and stable fixation of the AC without increasing the incidence of complications [22, 23]. However, patients without CC ligament augmentation in the current study had worse functional results before hook plate removal, a higher incidence of radiographic acromion osteolysis, and a higher incidence of peri-implant fracture. One possible reason for this finding is that additional CC ligament augmentation can share the stress loading around the AC joint. When the hook plate is used alone, the stress is concentrated on the two ends of the hook plate, which will lead to an increase in peri-implant fractures, as well as an increase in the incidence of acromion osteolysis.

In the current study, the incidence of peri-implant fractures (including distal clavicle fractures and acromion fractures) of the hook plate was higher in patients without CC augmentation (8/112) than in patients with augmentation (1/105); revision surgery was necessary for both fractures. For peri-implant distal clavicle fractures, changing to a longer plate can fix the AC separation, while also fixing the new fracture at the same time. However, the hook plate system is no longer feasible for peri-implant acromion fractures because the broken acromion cannot be used as a point of force application. In the current study, a closed-loop, double endobutton stabilization TightRope system (Arthrex, Naples, FL, USA) was used in the revision surgery for AC separation with acromion fractures.

The Constant-Murley score is often used to evaluate the shoulder function before and after hook plate removal for AC separation. In the current study, the average score was 46.3 before removal, 82.8 at 3 months after removal, and 90.5 at 6 months after removal. In the study of Kumar, the scores were 60.3 and 83.7 at 3 and 6 months after removal, respectively [33]. In comparison, the patients in our study had higher scores after removal, which may have been due to early removal of the hook plate and the early physical therapy protocol. We arranged implant removal after 3 months, and the average duration from surgery to removal was 102 days. Following implant removal, all patients immediately performed passive and active stretching exercises without restriction. Early aggressive ROM exercise can decrease the adhesion of the shoulder joint and may improve the functional outcome.

In current study, there was more significant acromion osteolysis in patients without CC augmentation (65/112) than in patients with augmentation (25/105). Furthermore, patients without osteolysis had better Constant-Murley scores than the patients with osteolysis before removal, but there was no significant difference after removal. A previous study of hook plate-related acromion osteolysis revealed that the incidence of acromion osteolysis was approximately 50%, and had no clear impact on the Constant-Murley score [34]. This conclusion was very similar to our results, although we demonstrated a lower incidence of acromion osteolysis in cases where CC ligament augmentation was performed.

The current study has several limitations. This was a retrospective study and the cases were not randomly distributed; thus, the possibility of selection bias should be considered in each group. We used the form of Constant-Murley score to evaluate all of the shoulder surgeries in our hospital, but we did not have the individual numeric value for each ROM measurement. Furthermore, we only took shoulder AP view, oblique view, scapula Y view, and chest radiographs to evaluate the AC separation; we did not take Zanca view, Alexander view, and weight-bearing stress views. In line with this, the horizontal displacement of AC separation was not evaluated. Although it is high risk for concomitant injuries in high grade AC separation [35], we did not perform MRI or CT regularly for all cases. Therefore, some concomitant injuries might be neglected. Moreover, there were many uncertain factors that influenced the results because the surgeries were not always performed by the same surgeon. Therefore, future studies should be designed with the aim to reduce multi-factor impacts, and efforts should be made to analyze long-time complications.

## Conclusion

The patients of Rockwood type V AC separation treated by hook plate without CC ligament augmentation had worse functional results before hook plate removal, a higher incidence of radiographic acromion osteolysis, and a higher incidence of peri-implant fractures than those patients with CC ligament augmentation. Therefore, CC ligament augmentation is highly recommended to improve short-term outcomes and decrease complications for Rockwood type V AC separation treated by hook plate.

## Data Availability

The datasets used and analyzed during the current study are available from the corresponding author on reasonable request.

## Abbreviations

AC: Acromioclavicular
CC: Coracoclavicular
AP: Anteroposterior
PA: Posteroanterior
VAS: Visual Analogue Scale
ROM: Range of motion
